# Oral Care during Pregnancy: Attitudes of Brazilian Public Health Professionals

**DOI:** 10.3390/ijerph9103454

**Published:** 2012-09-28

**Authors:** Renata Toledo Alves, Rosangela Almeida Ribeiro, Luciane Rezende Costa, Claudio Rodrigues Leles, Maria do Carmo Matias Freire, Saul Martins Paiva

**Affiliations:** 1 Estacio de Sa College, Avenida Presidente Joao Goulart, 600, Cruzeiro do Sul, Juiz de Fora, MG 30130-900, Brazil; Email: retoledojf@terra.com.br; 2 Department of Social and Child Dentistry, Faculty of Dentistry, Federal University of Juiz de Fora, Rua José Lourenço Kelmer, Bairro São Pedro, Juiz de Fora, MG 36036-900, Brazil; Email: ralmeida@powerline.com.br; 3 Department of Preventive and Restorative Dentistry, Faculty of Dentistry, Federal University of Goias, Primeira Avenida, Setor Universitario, Goiania, GO 74605-220, Brazil; Email: crleles@odonto.ufg.br; 4 Department of Stomatological Sciences, Faculty of Dentistry, Federal University of Goias, Primeira Avenida, Setor Universitario, Goiania, GO 74605-220, Brazil; Email: mcmfreire@yahoo.com.br; 5 Department of Pediatric Dentistry and Orthodontics, Faculty of Dentistry, Federal University of Minas Gerais, Avenida Antonio Carlos, 6627, Pampulha, Belo Horizonte, MG 31270-901, Brazil; Email: smpaiva@uol.com.br

**Keywords:** attitudes, health personnel, oral health, pregnancy

## Abstract

There is little information about health professionals’ behavior regarding oral health care during pregnancy. We evaluated attitudes of obstetricians/gynecologists, nurses, and dentists working at a public community service towards pregnant women’s oral health. Health professionals responded to a self-applied questionnaire. Cluster analysis identified two clusters of respondents; Chi-square, Student’s t test, and logistic regression were used to compare the two clusters in terms of the independent variables. Respondents were categorized into cluster 1 ‘less favorable’ (n = 159) and cluster 2 ‘more favorable’ (n = 124) attitudes. Professionals that had attended a residency or specialization program (OR = 2.08, 95% CI = 1.15–3.77, *p* = 0.016) and worked exclusively at the public service (OR = 2.15, 95% CI = 1.10–4.20, *p* = 0.025) presented more favorable attitudes. Obstetricians/gynecologists (OR = 0.22, 95% CI = 0.09–0.54, *p* = 0.001) and nurses (OR = 0.50, 95% CI = 0.29–0.86, *p* = 0.013) showed less favorable attitudes than dentists. Health care providers’ attitudes regarding pregnant women’s oral health were related to their occupation, qualification, and dedication to the public service.

## 1. Introduction

“Oral health care should be a goal in its own right for all individuals (…) public policies that support comprehensive dental services for vulnerable women of childbearing age should be expanded” [1]. To date, a large number of pregnant women in countries such as Greece [2], the United States [3], Australia [4], and Italy [5] have been unaware of the oral problems that may arise during the antenatal period and their consequences for mothers’ and infants’ oral and systemic health.

Several studies have been performed regarding knowledge, attitudes and practice in relation to oral health during pregnancy according to different health professionals. One survey [6] of 55 obstetricians conducted in North Carolina revealed some misconceptions on this topic, such as that tooth decay may worsen and women may or definitely lose teeth during pregnancy. Only half of those respondents recommended dental exams during pregnancy [6]. In Jordan, 88% of 250 physicians advised postponing dental treatment for after giving birth [7]. Other groups of American obstetrician-gynecologists, although they recognized the importance of good oral health during pregnancy, did not address it [8,9]. In India, gynecologists were divided on the acceptance of periodontal disease as a risk factor for pre-term low-birth-weight children [10]. A Brazilian study reported that, despite the fact obstetricians were aware of the association between gingival inflammation and adverse obstetric outcomes, their attitudes were not in agreement with the apparent knowledge regarding periodontal disease and its possible repercussions [11].

The opinions of nursing practitioners and certified nurse midwives on oral health care for pregnant women were also sought by the North Carolina research group [12]. Among 219 nurses, 86% reported referring patients for dental health screening; many showed equivocal information regarding the more favorable prenatal trimester to initiate dental treatment but admitted the need to collaborate with dental professionals to reduce risks during pregnancy [12].

Even dentists need specific education to provide proper oral care to pregnant patients, according to two studies with American general dentists from Oregon [13,14]. In one study [13], some dentists presented misinformation regarding routine procedures—obtaining radiographs, sedating with nitrous oxide, performing scaling and root planning, opening and broaching, or extracting a single tooth as an emergency service. Further investigations in the United States [8] and also in Brazil [15] showed that dentists and/or obstetricians diverged from scientific literature and among themselves on several recommendations related to dental care, e.g., local anesthetics, prenatal fluoride supplementation, and dental radiographs.

Although the aforementioned studies investigated some aspects of health care providers’ knowledge and opinions on oral health care during pregnancy, they lack information about other factors that could influence professionals’ attitudes besides their occupation. Agreeing with the inference that “attitudes are significant determinants of accurate knowledge and current practice” towards pregnant women’s dental care [14], we conducted a survey to evaluate the attitudes of obstetricians/gynecologists, nurses, and dentists from public primary health care units to the oral health aspects of pregnancy.

## 2. Methods

### 2.1. Participants

A cross-sectional survey was carried out involving 288 health professionals (obstetricians/ gynecologists, nurses, and dentists) working in all 110 primary health care centers of the Brazilian National Health System (SUS) in the city of Goiania during 2007. Goiania is the capital of the State of Goias, which is located in Middle West region of Brazil and has 1,256,514 inhabitants [16]. All 541 SUS health professionals (obstetricians/gynecologists, nurses, dentists) whose practice was focused on child-mother health were eligible and invited to participate in the study. Those on vacation, practicing administrative assignments, rendering services from other centers, or working in other institutions were excluded.

SUS consists of the set of actions and health services provided by federal, state, and local agencies and public institutions. It is based on the principles of universal access to health services, full assistance, and equity [17]. The study participants had part-or full-time jobs in SUS, where they worked in one of the two models of primary health care, namely the family health program (ESF) or conventional community service. The ESF model is a comprehensive care program focused on families and includes prevention, promotion, healing, and rehabilitation through team work [18]. In the conventional service, health professionals usually work on their own and are not supposed to integrate their practices with those of colleagues.

The Human Research Ethics Board of the Federal University of Goias approved this study, and participants read and signed the consent forms.

### 2.2. Data Collection

A questionnaire was developed to assess the dependent and independent variables of the study. Evidences from the literature helped in the development of the first version of the questionnaire, which was tested in a group of 15 professionals (three obstetricians/gynecologists, four nurses, and eight dentists). Then, the answers and comments from this group were read by two professors with expertise in questionnaire design and two Masters’ students for evaluation and refinement of the instrument. Changes were made in format and coding to improve clarity and reduce response burden.

The final version of the questionnaire was divided into two parts. The first part comprised 10 items regarding independent variables such as gender, occupation, professional formal education, and job activities. The second part measured the dependent variables through items relating to professionals’ attitudes towards pregnant women’s oral health. This part of the questionnaire included 14 items (first column, Table 1) answered according to a five-point Likert scale: ‘totally disagree = 1’, ‘disagree = 2’, ‘unconcerned = 3’, ‘agree = 4’, and ‘totally agree = 5’. Total scores could vary from 14 to 70. Higher scores indicated better attitudes and the respondents’ tendency to agree with the statements. The questionnaire’s reliability was estimated at the end of data collection (Cronbach’s Alpha Coefficient = 0.65), indicating acceptable internal consistency for this study.

Envelopes containing a cover letter, the informed consent form, and the questionnaire were delivered during a 4-month period to 541 professionals who had met the inclusion criteria. One researcher (RTA) informed the directors of each health care center about the study and asked for their help with envelope distribution and collection. In that first attempt, 142 forms were returned. Then the researcher visited each health unit to motivate participants and distributed the envelopes again to all gynecologists/obstetricians, nurses, and dentists who had not responded the first time. After one week, the researcher went back to all units and collected 146 more forms. No further attempts were made to increase the response rate.

### 2.3. Statistical Analysis

Data were analyzed using IBM SPSS Statistics Version 19. A two-step cluster analysis was used to define and evaluate the best grouping of subjects based on their similarities in responding to the 14 items on attitudes towards oral health during pregnancy. This method was data-driven, so it should be more valid than a subjective classification of the correlation among answers to the questionnaire. The two-step method is a one-pass-through-the-data approach which addresses the scaling problem by identifying pre-clusters in a first step, then treating these as single cases in a second step which uses hierarchical clustering, seeking to identify a set of groups which both minimize within-group variation and maximize between-group variation. In this study, the cluster analysis identified two clusters of health professionals (dependent variable), one with less favorable attitudes towards oral health during pregnancy (Cluster 1) and the other with more favorable attitudes (Cluster 2). The importance of each variable within each cluster was represented by a test in which each cluster group was tested against the overall group, sorted by the importance rank of each variable.

Chi-square and Student’s t test were carried out to compare the two clusters in terms of the independent variables. Logistic regression, forward likelihood ratio stepwise method, was used to determine the independent variables that accounted for the differences between professionals that had less or more favorable attitudes. The level of significance was set as α ≤ 0.05.

## 3. Results

Of the 541 questionnaires sent, 288 were completed and returned (53.2% response rate). The response rates for occupation were: obstetricians/gynecologists 33.3% (37 questionnaires), nurses 45.2% (142 questionnaires), and dentists 47.0% (109 questionnaires). Respondents’ total score for part 2 of the questionnaire varied from 34 to 70 (mean 53.1, SD 5.7). 

Cluster analysis excluded five out of the 288 cases and resulted in two groups: cluster 1 with less favorable attitudes (n = 159), total scores ranging from 38 to 61 (mean 50.0, SD 4.1); cluster 2 with more favorable attitudes (n = 124), total scores ranging from 43 to 70 (mean 57.2, SD 4.6). Table 1 depicts the relative importance of the variables in differentiating each cluster. The greater the t-test value, the greater the significance of the variable for the cluster formation, listed in descending order of relevance for the clustering process based on statistical significance.

**Table 1:** Mean scores and importance of variables in cluster formation.

	Mean score (95% CI)		Relative importance for the formation of clusters (t test)	
Variables	Cluster 1 ^a^ (less favorable)	Cluster 2 ^a^ (more favorable)	Cluster #1	Cluster #2
Periodontal disease influences the pregnant patient’s general health	4.0 (3.9–4.1)	5.0 (4.9–5.0)	−12.26	32.80
Dental caries influences the pregnant patient’s general health	4.0 (3.9–4.1)	4.9 (4.8–5.0)	−8.85	18.00
Prenatal follow-up should be multi-professional	4.5 (4.4–4.6)	5.0 (4.9–5.0)	−4.16	14.50
Periodontal disease during pregnancy has consequences for the fetus	3.5 (3.3–3.6)	4.3 (4.1–4.4)	−4.87	6.10
I always refer the pregnant patient to other health professionals	3.7 (3.6–3.9)	4.3 (4.1–4.5)	−3.42	3.70
Periodontal disease during pregnancy influences labor	3.0 (2.9–3.2)	3.7 (3.4–3.8)	−3.62	3.50
The etiological factors for dental caries are the same for pregnant and non-pregnant patients	2.8 (2.7–3.0)	3.5 (3.3–3.7)	−3.62	3.40
My knowledge on the oral health of pregnant women is totally satisfactory	2.8 (2.6–3.0)	3.4 (3.1–3.6)	−3.01	NS ^b^
I always inform the pregnant patient about oral health related aspects	3.8 (3.7–4.0)	4.3 (4.1–4.5)	NS ^b^	3.10
Pregnant women present greater risk for periodontal disease than non-pregnant ones	3.7 (3.6–3.8)	4.0 (3.9–4.2)	NS ^b^	NS ^b^
I feel totally prepared to assist the pregnant patient working in a team	3.8 (3.6–3.9)	4.1 (3.9–4.3)	NS ^b^	NS ^b^
My relationship with other health professionals regarding pregnancy care is totally satisfactory	3.4 (3.2–3.5)	3.6 (3.4–3.8)	NS ^b^	NS ^b^
The patient’s oral health is essential for normal pregnancy development	4.2 (4.1–4.4)	4.4 (4.3–4.6)	NS ^b^	NS ^b^
The etiological factors for periodontal disease are the same for pregnant and non-pregnant patients	2.8 (2.7–3.0)	3.0 (2.7–3.2)	NS ^b^	NS ^b^

^a^ Cluster 1 includes professionals with less favorable attitudes towards oral health during pregnancy and Cluster 2 includes professionals with more favorable attitudes towards oral health during pregnancy ^b^ NS—Variables not important for cluster formation

We compared the clusters for the independent variables (Table 2) through bivariate analysis and found that dentists had more favorable attitudes towards oral health during pregnancy compared to obstetricians/gynecologists and nurses (*p* = 0.026); also did health professionals who had received any information about oral health during formal education (*p* = 0.032).

**Table 2:** Association between attitudes towards oral health during pregnancy and variables.

Independent variables	Cluster 1 Less favorable attitudes, n (%)	Cluster 2 More favorable attitudes, n (%)	*p-*value^ c^
Sex			0.074
*Female*	121 (53.5)	105 (46.5)	
*Male*	38 (66.7)	19 (33.3)	
Occupation			0.026
*Obstetrician/Gynecologist*	25 (46.7)	11 (53.3)^ a^	
*Nurse*	84 (60.0)	56 (40.0)^ a^	
*Dentist*	50 (69.4)	57 (30.6)^ b^	
Work exclusively at the public service **			
Yes	117 (52.9)	104 (47.1)	0.051
*No *	39 (67.2)	19 (32.8)	
Time after graduation (years), mean ± SD	15.2 ± 9.1	13.9 ± 7.6	0.205
Specialization or residency **			0.063
Yes	109 (52.9)	97 (47.1)	
*No *	49 (65.3)	26 (34.7)	
Time working at the Brazilian National Health System (years), mean ± SD	8.1 ± 7.3	9.5 ± 7.6	0.121
Type of primary health care center			0.256
*Conventional*	117 (58.2)	84 (41.8)	
*Family Health Program*	39 (50.6)	38 (49.4)	
Major number of patients are pregnant			0.947
* Yes *	21 (55.3)	17 (44.7)	
* No *	129 (55.8)	102 (44.2)	
Pregnant patients are referred from other health professionals			
* Yes *	32 (51.6)	30 (48.4)	0.225
*No *	107 (60.5)	70 (39.5)	
Content “oral health during pregnancy” in the professional formal education			
* Yes* **	82 (50.3)	81 (49.7)	0.032
*No*	68 (63.6)	39 (36.4)	

^a,b ^Distinct letters mean that that occupation significantly differed from the others in the same cluster ^c ^Chi-square test or Student’s t test Results in bold type significant at 5% level

In the logistic regression analysis, all independent variables were initially included in the model, but only three remained in the final model (Table 3). Professionals that had attended a residency or specialization program as well as worked exclusively at the public service presented increased odds of having more favorable attitudes. Obstetricians/gynecologists and nurses had increased odds for less favorable attitudes than dentists.

**Table 3:** Final multiple logistic regression model for the independent variables explaining more favorable attitudes towards oral health of pregnant women.

Independent variable	Category	OR (95% CI)	*p*-value
Occupation	Dentist	1	
	Nurse	0.50 (0.29–0.86)	0.013
	Obstetrician/gynecologist	0.22 (0.09–0.54)	0.001
Specialization or residency	No	1	
	Yes	2.08 (1.15–3.77)	0.016
Work exclusively at the public service	No	1	
	Yes	2.15 (1.10–4.20)	0.025

Correctly predicted% = 62.7; Nagelkerke R^2^ = 0.106

## 4. Discussion

Oral health care during pregnancy cannot be dissociated from systemic health, and it means more than discussing if periodontal disease is a causative factor for preterm birth/low birth weight. It represents a comprehensive and multidisciplinary approach aiming to empower women’s wellness, then allowing people to understand the importance of caring for their oral and systemic health, from educational measures to pain control and oral disease treatment. From this perspective, three major findings arose from this study: 1. Nurses and obstetricians/gynecologists were less prone to favorable attitudes towards women’s oral health, compared to dentists; 2. Health professionals that work exclusively at the public service or had attended post-graduation programs (residency or specialization) reported more favorable attitudes; 3. Pregnant women’s oral health was a poorly understood topic for this group of health professionals.

The clusters generated by the respondents’ attitudes highlighted that professionals categorized as having ‘more favorable attitudes’ presented a more positive approach in understanding the connection between oral and systemic health and the need for a multi professional team in pregnancy care. In a cultural context where the physician’s opinion about this subject is more valued by lay people than the dentist’s [19], we should emphasize the importance of a multidisciplinary approach to pregnant women. Interestingly, another study showed that although obstetricians are aware of the potential role of periodontal disease as a pregnancy risk factor, few incorporate dental care into their clinical medical practice [6]. They are probably unaware of dental procedure safety during pregnancy, as found in another report [9].

In the bivariate analysis, being a dentist was significantly associated with more favorable attitudes, compared to obstetricians/gynecologists and nurses, and this significance was maintained in the regression model. By converting odds ratios for obstetricians/gynecologists and nurses, we verify that they have 4.6 and 2.0 increased odds of less favorable attitudes, respectively, compared to dentists. This is an important finding because when we consider team work we should not encourage competition based on workers’ knowledge and practice; every professional should understand his/her role in a group. Although oral health education is one of the basic roles of dental professionals, other health workers should not miss opportunities to contribute to oral health promotion. There is evidence that pregnant women do not usually seek dental assistance during their gestational period [2,20,21]. Professionals involved in prenatal care should discuss the importance of oral health with pregnant women and refer patients to dental treatment when necessary [7]. The importance of self-care to each member of the healthcare team should be stressed, since they are the disseminators of knowledge to those under their care, as previously stated [11]. Promoting oral health during pregnancy can improve maternal oral health, reduce the risk of early caries development in children and positively influence the behaviors and attitudes of mothers and their children in relation to oral health [22].

Professionals who attended a specialization or residency program had 2-fold increased odds of more favorable attitudes. This was an expected finding, because in a global sense, the predoctoral/ undergraduate curricula of non-dental health care professions (medicine, nursing, and pharmacy) do not contain adequate content related to oral-systemic health [23,24,25]. Besides, our findings showed that professionals who did not have the content “oral health during pregnancy” in their formal education presented less favorable attitudes. Also, if we consider that to be an obstetrician/gynecologist in Brazil the doctor must attend a residency program, we can hypothesize that this kind of residency programs lacks the oral health subject. Another study observed that, according to patients’ reports, health professionals disseminate and strengthen misconceptions and fears about dental care and oral health during pregnancy [19]. Our findings support the need for investment in education on oral health care during pregnancy, both at undergraduate and graduate level, as other studies have advocated [6,12,19]. 

In this study, professionals that work exclusively in SUS had 2-fold increased odds of more favorable attitudes. This might be because they have more time to dedicate to and understand the public service process and they work in multi professional teams. A part-time job in SUS may not allow health professionals such as dentists to have an in-depth knowledge of SUS principles, or they may have an awareness of the principles but also be skeptical about them [26]. However, our finding that professionals who integrated family health programs (ESF) did not differ in their attitudes from those who worked in the conventional model does not support this last hypothesis. A more comprehensive assessment of the ESF could elucidate this issue.

Even though the cluster analysis revealed two natural groupings within our data, the general scores for attitudes favoring oral health during pregnancy were fair. Beside the knowledge issues discussed before, we cannot deny that professionals perceive some service barriers that might have influenced their answers. Australian midwives, for example, were reluctant to discuss oral health with pregnant women because of a lack of appropriate referral pathways to the Public Dental Services, time, and required competencies [27]. Also, American dentists perceived barriers which were associated with providing fewer dental services to pregnant women: time, economics, skills, dental staff resistance, and peer pressure [14]. This should be further investigated.

This study has some limitations. The questionnaire was not formally validated; instead, it was tested in a pilot study to determine the understanding of the health professionals, and the final sample reached a moderate internal consistency. Although the sample size was sufficient for the purpose of our investigation and similar to the correspondent literature, the rate for non-response should not be disregarded, especially for the physician group. We could not analyze non-response bias because we did not have enough information about non-respondents. In a similar study carried out in the Brazilian public health system, the lack of time reported by professionals and the difficulties researchers found in the distribution of questionnaires were felt to contribute to the low response rate usually achieved [28]. 

All in all, to properly manage health during pregnancy, health care providers must improve their attitudes to the link between oral and systemic health. Undergraduate and graduate curricula for health sciences programs should encourage opportunities for learning in this area in theoretical and real scenarios, having students working in multidisciplinary teams. Public service strategies should support health professionals in achieving this goal.

## 5. Conclusions

This survey showed that ‘dentist’ health professionals, those who have worked exclusively in the public service or had attended post-graduation programs (residency or specialization) reported more favorable attitudes towards the oral health aspects of pregnancy.
